# Diphtheria of the Skin

**Published:** 1909-02-13

**Authors:** 


### Dermatology.

### DIPHTHERIA OF THE SKIN.

It has long been known that diphtheria may affect the skin in the form of diphtheritic membranes upon pre-existing sores or wounds. Such cases were described by Trousseau, and examples are from time to time reported in the journals. Hilton Fagge insisted upon the rule that blisters should never be ordered for a patient suffering from diphtheria, on account of the liability to the subsequent development of false membranes upon the areas to which the blisters were applied. In some cases of diphtheritic infection of the skin the membrane formation has been extensive, and many cases have been fatal. A second form of diphtheria of the skin is that known as diphtheritic whitlow, sometimes seen in doctors or nurses.

Recently a still more interesting form of skin lesion has been recognised—namely, an eruption which simulates impetigo or ecthyma, and so closely that it has been only by accident that its real nature has been discovered. Now that such cases are recognised it is highly probable that they will be found to be of not infrequent occurrence. The first of these cases was recorded by Labbé and Demarque in France in 1905. Briefly, the cases were as follows:—

*Case* 1.—A child, aged four years, with an impetiginous and ecthymatous eruption, disseminated and generalised. On the trunk and thighs there were rounded crusted patches and rounded sharply-defined ulcers. The throat was red, but there was no membrane. The temperature was normal and the general condition good.

*Case* 2.—An infant, aged two years and five months. There was impetigo of the scalp and face—vesicles and crusted lesions—and a large excoriated area around the left ear; the lips were excoriated, and there was a crusted patch on the arm. The child died from pneumonia.

Both these cases had suggested ecthymatous impetigo, but the obstinacy of the lesions to treatment and their long duration led to their more careful study, and on making cultures, in addition to a staphylococcus, Lœffler's diphtheria bacillus was found. The first child recovered completely with antitoxin.

A case very similar to these two was reported in 1907 by Schucht in Germany. The patient was a boy, aged three years, and he presented the clinical picture of infantile ecthyma. On the thighs, buttocks, and forearms and behind the right ear were sharply circumscribed ulcers. The primary lesion was found to be a blister, which broke and gave rise to a superficial ulcer. Lœffler's bacillus was found in the lesions.

In 1908 three cases were reported in this country. The first of these—Slater's case—was a girl, aged thirteen years. For three years she had suffered from a wide-spread vesicular eruption upon the face, scalp, ears, neck, shoulders, vulva, abdomen, and thighs. This condition, obstinate to ordinary local treatments, at once cleared up with antitoxin injections. Lœffler's bacillus and staphylococcus were obtained in cultures from the lesions. The second case was recorded by Eddowes and Hare. The patient was a girl, aged eleven years. Bullæ were present on the arms, the hands, and the lips; on the left anterior pillar of the fauces was a ruptured and healing vesicle. The general condition was bad, and the patient was admitted to hospital on that account. Cultures were made and Lœffler's bacillus was found, together with staphylococcus and streptococcus. Recovery was rapid without antitoxin.

A third case, "a case of diphtheria associated with impetigo," was reported by T. P. Puddicombe. The patient was a boy, aged eight years. He had impetigo of the face of six days' duration, and a congested throat with a patch of membrane on one tonsil, and nasal discharge of one day's duration. The temperature was 99.5°, and the boy did not appear ill. A virulent strain of Klebs-Lœffler's bacillus was found both in the throat and in the impetigo lesions. Anti-diphtheritic toxin was given, and the case ran a normal course to recovery.

It seems probable from these cases that the bacillus of diphtheria is capable of giving rise on the skin to vesicular or bullous lesions, which may dry up to form crusts resembling those of impetigo, or lead to excoriations and more or less deep ulcerations suggesting ecthymatous lesions. At present, however, these cases can only be regarded as probably of diphtheritic origin, for the finding of Lœffler's bacillus in the lesions cannot be claimed as proof of their diphtheritic nature. Nor does the fact that some of the cases have improved rapidly with antitoxin decide the matter, for it is well known that extensive eruptions of ordinary impetigo or of impetiginous eczema will clear up under similar conditions—*e.g.* on the administration of horse serum, during vaccination, after injection of tenanus antitoxin, etc. So far no case has been reported in which these eruptions have been followed by diphtheritic paralysis.

